# The Reorganization Indifferences—Letter from Dr. Shropshire

**Published:** 1902-08

**Authors:** Walter Shropshire

**Affiliations:** Yoakum, Texas


### The Reorganization Indifferences—Letter from Dr. Shropshire.

YOAKUM, TEXAS, June 2, 1902.

*Editor Texas Medical Journal, Austin, Texas.*

DEAR SIR: Your editorial in the last issue of the JOURNAL on the plan of organization prompts me to make some suggestions relative to the plan alike cussed and discussed at Dallas.

There seems to prevail a belief that the plan of districting the State has for its object the curtailing of membership in certain large societies now existing, namely, the North Texas and the South Texas Medical Associations, and to force some members into districts not convenient to them; also that the districts—mere suggestions in the minority report—are to be the lines, permanent and unchangeable; all of which is foreign to the desire and intention of the promoters of the plan.

The plan is to divide the State into fifteen or twenty districts and to organize a district association in each one where there is no society at present which will reorganize upon a plan compatible with the State Association, thus making the district associations the portals for entrance to the Texas State Medical Association instead of the county society as other States have done. The purpose of this plan is the thorough, uniform and systematic organization of the whole State, omitting no portion thereof.

While it should restrict each member's entrance to the Texas State Medical Association to the district association where he resides, it would in nowise prohibit his membership in any other district association or independent society. In fact, it is the desire of the advocates that each district association make provision to receive as members residents of different districts; provided, first, that the individual is a member of his own district association; second, that he be not counted in a basis for representation in the House of Delegates of the State Association, the latter provision to prevent a double representation, one in each district association.

The matter of placing membership in inconvenient districts had no part in the desires of the promoters of the plan, and they agree that provisions should be whereby the physicians of any county may be changed from their residence district to a more convenient one, when two-thirds of the physicians in the county sign petition to the House of Delegates for the change and the petition is granted. Such a provision is necessary even were there no dissatisfaction at the time the divisions are made, for the building of new roads and means of travel will alter conditions so that in many cases change of district will afford convenience.

Relative to the districts outlined, which were mere suggestions, it is evident that they might be much improved when members from all parts are consulted as to means of transportation; for no committee can in a few hours district the State from the map only, to the best advantage.

The plan of districting the State and making the district association the unit of the State Association is the only one that can be adopted in our State which will be thorough and practical, for to make the county society the base (as in New York and other States) would rule out of the State Association all physicians in counties too sparsely populated to support a county society.

The suggestion so often made that some of our larger societies will not submit to the plan of reorganization is absurd, for some of our best State Association members belong to these very societies and are heart and soul for reorganization upon the most thorough and effective plan; these men will shape the action of their societies to meet the best interests of the profession, no matter if some of the members, not State Association men, do not want to be brought in. Even if an existing society refused to become a branch of the State Association, there would be organized in that district an association in conformity with the State Association, through which all who desired could become members of the State Association, and the old society might continue independently without injury to or from the district association.

Contrary to your opinion, Mr. Editor, there is and can be no sectional strife over the reorganization; it is only a question with us all how to make it most thorough and efficient, and the association to a man will abide by the decision reached by a majority vote. [They refused to do so at Dallas.—EDITOR.]

I am ready to wager you, Mr. Editor, a "small Budweiser" that not a man who has carefully read the report of the American Medical Association's reorganization and the two reports ordered published and distributed will advocate that conglomeration you and I helped to bring out of the committee selected to act on the plans of reorganization at San Antonio. Is it a wager?

Fraternally,

WALTER SHROPSHIRE.

